# Patients’ and providers’ perspectives on medication relatedness and potential preventability of hospital readmissions within 30 days of discharge

**DOI:** 10.1111/hex.12993

**Published:** 2019-11-16

**Authors:** Elien B. Uitvlugt, Marjo J. A. Janssen, Carl E. H. Siegert, Anna J. A. Leenders, Bart J. F. van den Bemt, Patricia M. L. A. van den Bemt, Fatma Karapinar‐Çarkit

**Affiliations:** ^1^ Department of Hospital Pharmacy OLVG Amsterdam The Netherlands; ^2^ Department of Internal Medicine OLVG Amsterdam The Netherlands; ^3^ Department of Pharmacy Sint Maartenskliniek Nijmegen The Netherlands; ^4^ Department of Pharmacy Radboud University Medical Centre Nijmegen The Netherlands; ^5^ Department of Hospital Pharmacy Erasmus MC University Medical Center Rotterdam Rotterdam The Netherlands

**Keywords:** hospital readmissions, medication, patients’ perspectives, preventability, providers’ perspective

## Abstract

**Background:**

Hospital readmissions are increasingly used as an indicator of quality in health care. One potential risk factor of readmissions is polypharmacy. No studies have explored the patients’ perspectives on the medication relatedness and potential preventability of their readmissions.

**Objective:**

To compare the patients’ perspectives on the medication relatedness and potential preventability of their readmissions with the providers’ perspectives.

**Methods:**

Patients unplanned readmitted within 30 days after discharge at one of the participating departments of OLVG Hospital in Amsterdam were interviewed during their readmission. Patients’ perspectives regarding medication relatedness of their readmissions, the potential preventability, possible preventable interventions, and satisfaction with medication information were examined. Health‐care providers also reviewed files of these readmitted patients. Primary outcome was the percentage of medication‐related and potentially preventable readmissions according to the patient vs the provider. Descriptive data analysis was used.

**Results:**

According to patients, 36 of 172 (21%) readmissions were medication‐related, and of these, 21 (58%) were potentially preventable. According to providers, 26 (15%) readmissions were medication‐related and 6 (23%) of these were potentially preventable. Patients and providers agreed on the medication relatedness in 11 of the 172 readmissions, and in two of these, agreement on the potential preventability existed. According to patients, preventive interventions belonged mostly to the hospital level, followed by the primary care level and patient level.

**Conclusion:**

Patients and providers differ substantially on their perspectives regarding the medication relatedness and preventability of readmissions. Patients were more likely to view medication‐related readmissions as preventable.

## 1 INTRODUCTION

Unplanned hospital readmissions within 30 days are increasingly used as an indicator of quality and safety in health care.^1^, ^2^, ^3^ This assumed that readmissions are preventable. Measuring the preventability of readmissions is a challenge, because uniform factors related to preventable readmissions and a clear definition of ‘preventability’ have not been established.^4^, ^5^, ^6^, ^7^ Feigenbaum et al^8^ found that on average, 8.7 factors contributed to each potentially preventable readmission. Those factors frequently occurred during follow‐up care and were related to transition care planning and care coordination. Medication management was a factor in more than a quarter of readmissions, including medication errors during or after index admission and inadequate patient and caregiver understanding of medication management. The existing literature on medication‐related readmissions shows that a median of 21% of readmissions are due to medication and 5%‐87% (median 69%) of these readmissions were deemed preventable.^9^ The risk for medication‐related problems increases with polypharmacy. A review indicates that 18%‐38% of patients report medication‐related problems after hospital discharge.^10^

As the patient is the only constant factor in the care continuum, information from the patient is needed to get insight into medication‐related problems occurring between discharge from hospital and readmission. Kari et al^11^ show that patient involvement is essential in detecting medication‐related problems, because otherwise poor therapy control, non‐optimal medication use, or intentional or unintentional non‐adherence might be missed.

However, studies investigating patients’ perspectives on medication relatedness and preventability of these readmissions are lacking. Consensus between patients and providers with respect to the role of medication as a potential cause of readmissions is necessary to achieve optimal pharmacotherapy.^12^ If a readmission is caused by medication according to the patient without being aware that his provider is not convinced of a causal association, a patient could stop independently with the suspicious medication resulting in non‐adherence. On the other hand, if a provider believes the readmission is caused by medication but the patient is unaware of the provider's perspective, medication could still be taken by the patient resulting in a repeated readmission.

First, the aim of this study is to describe patients’ perspectives on the medication relatedness and potential preventability of their readmissions and compare these with providers’ perspectives. Secondly, we describe the patients’ perspectives regarding interventions that could have prevented medication‐related readmissions and the patients’ satisfaction with information about medication during the index admission.

## 2 METHOD

### 2.1 Design and setting

The data for this cross‐sectional observational study were collected within the context of a larger study on all‐cause readmissions. This current study however focuses on medication‐related readmissions. The study was performed at OLVG, a general teaching hospital in Amsterdam, the Netherlands, from July 2016 until May 2017. A list with readmissions within 30 days of discharge was generated within the hospital information system and daily screened by the research coordinator for eligibility.

Patients ≥18 years readmitted within 30 days after an index admission (first admission) to one of the departments of cardiology, gastro‐enterology, internal medicine, neurology, psychiatry, pulmonology and surgery were interviewed during their readmission. Patients were excluded if they were transferred to another hospital or self‐discharged, or when it was not the first readmission of the patient and if the readmission was due to attempted suicide or when the patient did not use any medication at all. Furthermore, a readmission was excluded if it was scored by providers (see below) as unrelated to the index admission. This was done to exclude 30‐day readmissions that occurred coincidentally. For example, a patient admitted with pneumonia discharged in a good clinical condition and readmitted within 30 days due to a traffic accident. Finally, providers had access to the interviews and registered whether they had used the interview in their review to assess the preventability of a readmission. If a patient interview was used by providers, this interview was excluded as well. The study was approved by the local review board of the hospital (ACWO‐MEC, registration number: 16‐028). Patient data were obtained and handled in accordance with privacy regulations.

### 2.2 Pharmaceutical care during the index admission

In the OLVG Hospital, two different processes are carried out to improve continuity of pharmaceutical care.^13^

- On the departments of cardiology, pulmonology, internal medicine, gastroenterology and neurology, our hospital has implemented a Transitional Pharmaceutical Care (TPC) programme.^14^ In short, hospital pharmacy teams perform medication reconciliation at hospital admission and discharge using the dispensing history of the community pharmacy and information from the patient/carer himself. Any discrepancies between a patient's actual medication use and the medication prescribed in hospital are discussed with the resident. No formal medication review is performed. However, obvious errors in the pharmacotherapy are eliminated, for example lack of a laxative when an opioid is prescribed or no indication for hypnotics at discharge, addressing a stop date for antibiotics or opioids. The reason for medication changes is explained to the patient during discharge counselling, and a written medication summary is provided. The pharmacy team makes a TPC‐medication overview that the resident could upload into the discharge letter.
- On the departments of psychiatry and surgery, residents and nurses are responsible for assessing a patient's actual medication use by interviewing patients/carers. If regarded necessary, they can request the hospital pharmacy to obtain a dispensing history from the community pharmacy. At hospital discharge, the resident uploads information from the hospital's prescribing system or types information into the discharge letter to the general practitioner.

### 2.3 Patients’ perspectives

Patients were interviewed during their readmission, or three attempts were made by phone in case the patient was already discharged or when a caregiver (family member or partner) needed to be approached, or in case of a language barrier or when the patient was unable to answer the questions. A structured interview guide was developed based on previous studies on readmissions and expert opinion.^15^, ^16^, ^17^, ^18^, ^19^, ^20^ For the purpose of this study, the following main topics were included: patients’ perspectives on medication relatedness, patients’ perspectives on potential preventability and preventive interventions, and patients’ perspectives on medication‐related information received during index admission (File S1). Additionally, the following socio‐demographic factors were asked: nationality, living situation, educational level and self‐experienced health status. Format of the questions included multiple‐choice, yes/no and free text. Interviews were conducted by medical students who received the interview guide and were trained for this. Interviews lasted approximately 30 minutes. During the entire interview period, students were supervised by the coordinating physician‐researcher. Interviewers manually recorded responses on data extraction sheets in Access 2010 (Microsoft).

### 2.4 Providers’ perspectives

Health‐care providers who reviewed the readmissions were residents of the participating departments and a pharmacist. First, providers reviewed complete medical records of the readmitted patients to assess whether the readmissions were clinically related to the index admissions. If it was clinically related, the medication relatedness, using the algorithm of Kramer et al,^21^ and the preventability, using a modified algorithm of Schumock et al, were assessed.^22^ Readmissions that were assessed as potentially preventable by the providers or raised questions after the research coordinator's check were included to be discussed once a month, during a multidisciplinary meeting with the research coordinator, residents and a pharmacist. All readmissions assessed as medication‐related by the residents and pharmacist have been reassessed by a senior physician (CS) and a clinical pharmacologist (MJ) to validate the findings.

### 2.5 Outcomes

Primary outcome was the percentage of medication‐related and potentially preventable readmissions according to the patient vs the provider. Secondary outcomes were patients’ perspectives regarding interventions that could have potentially prevented the readmission and percentage of patients who were satisfied with information about medication during their index admission.

### 2.6 Data analysis

Quantitative analysis was performed in SPSS version 21.0 (IBM SPSS). Data from interviews were analysed in MS Excel 2010 (Microsoft). For each question, frequency tables were made. The content of the open questions was qualitatively (inductively) independently coded by EU and AL. The codes were compared and discussed until consensus was reached. Hereafter, both researchers placed the codes into categories, which were also discussed until consensus was reached. Each answer was classified in one of these categories and presented in frequency tables in MS Excel. Patients’ and providers’ perspectives on medication relatedness and potential preventability were compared descriptively.

## 3 RESULTS

**Figure 1:**
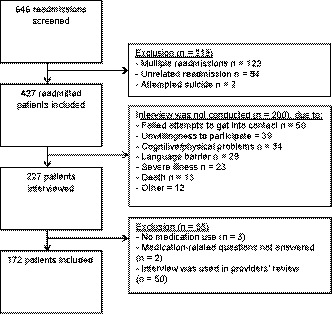
Flow of patients

Of 646 readmissions that were screened, 427 (66%) readmissions met the inclusion criteria, and 227 interviews were conducted, of which 172 (76%) were included in the final data analysis (Figure 1). Main reasons that the interview was not conducted were as follows: failed attempts to get into contact (n = 50), unwillingness to participate (n = 39) and cognitive/physical problems (n = 34). One hundred fifty interviews (87%) were conducted with patients, 4 (2%) with patients and caregivers and 18 (10%) with caregivers. The mean age of the included patients was 62 years (SD 18), 47% were male, and the mean number of the medications at discharge of index admission was 9.2 (SD 5.9) (Table 1).

**Table 1:** Patient and admission characteristics

Patient characteristics	n = 172
Interviewee	
Patient, n (%)	150 (87)
Patient and caregiver, n (%)	4 (2)
Caregiver, n (%)	18 (10)
Age, mean years (SD)	62 (18)
Male, n (%)	81 (47)
Native Dutch, n (%)	110 (64)
Living situation alone, n (%)	77 (45)
Help with medication use, yes (%)	64 (37)
Education level	
Primary (0‐8 y of education), n (%)	33 (19)
Secondary (9‐12 y of education), n (%)	88 (51)
Higher (>12 y of education), n (%)	49 (28)
Unknown, n (%)	2 (1)
Experienced health status	
Moderate/bad, n (%)	63 (37)
Good, n (%)	106 (62)
Missing, n (%)	3 (2)
Number of medicine at discharge (index admission), mean (SD)	9 (6)
Admission characteristics	
Length of stay, days, mean (SD)	6 (7)
Time between discharge and readmission, mean (SD)	12 (8)
Unplanned index admission, n (%)	139 (80)
Discharge department, n (%)	
Surgery	42 (24)
Pulmonology	36 (21)
Internal medicine	32 (19)
Cardiology	30 (17)
Gastroenterology	17 (10)
Neurology	15 (9)
Psychiatry	0 (0)

### 3.1 Patients’ and providers’ perspectives on medication relatedness and potential preventability

Table 2 shows patients’ and providers’ perspectives on medication relatedness and potential preventability in 172 readmissions. According to patients’ perspectives, 36 (21%) readmissions were medication‐related, of which 21 (58%) were potentially preventable (File S2). The causes (n = 23) of the potentially preventable readmissions according to patients were as follows: issues with dosage (n = 8, 35%), for example antibiotic discontinued too soon or too high dosage prescribed; change in medication (n = 6, 26%), for example medication changes that were unclear to the patient; a medication interaction (n = 1, 4%); costs (n = 1, 4%); or adherence (n = 1, 4%). In six readmissions (26%), the patient described an adverse drug reaction as a cause, but in most of those cases, the patient could not pinpoint which medication exactly was responsible for the side‐effects. According to providers’ perspectives, 26 (15%) readmissions were medication‐related, of which 6 (23%) were potentially preventable. In 11 of the 172 readmissions, patients and providers agreed on the medication relatedness, and in two of these, agreement on the potential preventability existed (File S2).

**Table 2:** Patients’ and providers’ perspectives on medication relatedness and potential preventability

	Patients’ perspectives				
Providers’ perspectives	Total readmissions (n = 172)		Not medication‐related (n = 136)	Medication‐related (n = 36)	
				Not preventable (n = 15)	Potentially preventable (n = 21)
	Not medication‐related (n = 146)		*121*	9	16
	Medication‐related (n = 26)	Not preventable (n = 20)	12	***5***	**3**
		Potentially preventable (n = 6)	3	**1**	***2***

Bold values show the number of readmissions with agreement on the medication relatedness. Italic values show the number readmissions with agreement on the medication relatedness and preventability between patients and providers.

### 3.2 Patients’ perspectives on preventive interventions

Of the readmissions that were medication‐related and potentially preventable according to the patient (n = 21), patients reported 23 preventative interventions. Hospital‐based interventions were 18 times reported, including performing more diagnostics (33%), improving medication‐related information (17%), providing a longer hospital stay (17%), treating symptoms/complaints (17%), providing better aftercare (11%) or reacting faster (6%) (Table 3). In two cases, patients reported that general practitioner–based interventions could have prevented the readmission, by reacting faster. Two patients reported that he or she could have prevented the readmission by being adherent to therapy.

**Table 3:** Patients’ reported interventions for preventable medication‐related readmissions (n = 21). Patients could mention more than one intervention

Question	Yes, n (%)
All interventions	23
Hospital‐based:	18 (78)
More diagnostics	6 (33)
Example patient's answer	
‘I did not get enough medicines to get an adequate INR I asked to monitor my blood, however this was not done. I got discharged with an INR of 1.2’.	
Improving medication‐related information	3 (17)
Example patient's answer	
‘I was confused about my diuretics, one was started and one was stopped. I would get some diuretics upon discharge, however at discharge there was a lot of confusion and I did not get them. Not taking the diuretics could contribute to my rehospitalisation’	
Longer hospital stay	3 (17)
Example caregiver's answer	
‘My father was discharged too early. The neurologist could not find anything and he thought it was something with the heart. However, the cardiologist refused to examine my father, so there was no follow‐up. We thought something was wrong with the medication, but they did not listen to us. Now he is readmitted due to a way too low blood pressure’	
Treating symptoms/complaints	3 (17)
Example patient's answer	
‘The anti‐inflammatory medicines should have been given longer, then the shortness of breath might not have come back’	
Better aftercare	2 (11)
Example patient's answer	
‘I read in the package leaflet that ciprofloxacin could cause pain in the Achilles tendon; I needed home care because I could not walk anymore because of the pain’	
React faster	1 (6)
Example patient's answer:	
‘I should have gotten a higher dose of dexamethasone earlier, then my readmission might have been prevented’	
General practitioner–based:	2 (9)
React faster	2 (100)
Example patient's answer:	
‘My general practitioner should have arranged home care, because I needed help with daily self‐care activities’	
Patient based:	2 (9)
Therapy compliance	2 (100)
Example patient's answer:	
‘I have mixed up Oxynorm^®^ and Oxycontin^®^’	
Other:	1 (4)
Unclassifiable due to lack of clear information	1 (100)

### 3.3 Patients’ satisfaction on medication‐related information

Table 4 shows patients’ satisfaction on medication‐related information. In readmissions that were medication‐related but not preventable according to patients’ perspectives (n = 15), patients reported in 93% (n = 14) that they had received as much information as they needed about medicines compared with 67% (n = 14) in readmissions deemed potentially preventable (n = 21). Also, information about side‐effects of medicines was more often scored as ‘as much information as I needed’ in readmissions not preventable according to patients’ perspectives compared with potentially preventable readmissions, 87% (n = 13) vs 43% (n = 9), respectively. In 73% (n = 11) of the readmissions scored as not preventable, patients received written instructions, compared with 57% (n = 12) in readmissions scored as potentially preventable.

**Table 4:** Patients’ satisfaction of medication‐related information during index admission

	Not medication‐related according to patients’ perspectives (n = 136), n (%)	Medication‐related according to patients’ perspectives (n = 36), n (%)	
		Not preventable (n = 15)	Potentially preventable (n = 21)
How much information did you receive during hospitalization about medicines you had to take at home?			
No information or some information, but not enough	19 (14)	1 (7)	7 (33)
As much information as I needed	117 (86)	14 (93)	14 (67)
How much information did you receive during hospitalization about side‐effects of medicines you had to take at home?			
No information or some information, but not enough	58 (42)	2 (13)	12 (57)
As much information as I needed	78 (58)	13 (87)	9 (43)
Did you receive written instructions at discharge about medicines you had to take at home?			
No	59 (43)	4 (27)	9 (43)
Yes	77 (56)	11 (73)	12 (57)

## 4 DISCUSSION

This study shows that according to patients, readmissions are more often medication‐related (21% of readmissions in patients vs 15% in providers) and are more often potentially preventable (patients 58% vs providers 23%) compared to providers. Patients and providers agreed on the medication relatedness in 11 of the 172 readmissions, and in two of these, agreement on the potential preventability existed. Patients reported most often that actions in the hospital were needed to potentially prevent readmissions. Patients who stated that their readmission was preventable more often reported that they lacked information regarding medicines and about side‐effects and written instructions.

To our knowledge, this is the first study showing the perspectives of patients and providers on the role of medication in readmissions. Previous studies have described the perspectives of patients and providers on the preventability of all‐cause readmissions.^8^, ^23^, ^24^, ^25^, ^26^, ^27^, ^28^ A recent European study investigated the opinions of all‐cause readmitted patients, their carers, nurses and physicians on predictability and preventability.^27^ They found that consensus on predictability and preventability of all‐cause readmissions was poor, especially between patients and professionals (kappa values ranged from 0.105 to 0.173). This is in line with our study, where patients reported more often that the readmissions were preventable compared to providers. Also, Smeraglio et al^24^ found that patients often felt more could have been done at discharge to prevent readmissions compared to providers. Interestingly, they found that nurse case managers more often agreed with the patients’ perspectives compared to physicians. They hypothesized that fundamentally, physicians place more onus on patients to self‐advocate for care, while nurse case managers emphasize the system providing support. This suggests that including the perspectives of the nurse case managers could be useful to assess the preventability from a broader perspective, including the help that the care system could have offered.

Several explanations can be given for the disparities in perspectives of patients and providers. First, this can be related to the differences in pharmacological knowledge between patients and providers. When providers review the readmission, they may recognize a complication or contraindication from a medication responsible for the readmission which the majority of patients would be unaware of. For example, if a patient is readmitted because of symptoms of a digoxin intoxication, a patient could think this is because of worsening of the underlying disease, where a provider will relate this to digoxin. Secondly, providers used the information available in the hospital to review readmissions and lack information about what happened after discharge. Therefore, medication‐related problems and compliance issues after discharge could be missed. Consequently, providers could relate the readmission to worsening of the underlying disease and patients could indicate that this is caused by medication‐related problems. Lastly, patients and providers differed in the perspective of the care that was needed. Some patients were dissatisfied at discharge because of different expectations of their admission and the continuation of care after discharge, while according to the providers, adequate standard of care has been provided. All in all, more studies are needed to identify the exact reasons for the gap between patients’ and providers’ perspectives.

Given the preventive interventions cited by the patients in this study: diagnostics, longer hospital stay, treating symptoms and improving medication‐related information, the patients seem to feel not ready for discharge. Van Galen et al^27^ showed that the patient reporting not feeling ready for discharge was strongly associated with predictability and preventability. Also, patients in our study who stated that their readmission was preventable were less satisfied about the information regarding medicines. Nowadays, patients are discharged as early as possible. As a consequence, it is a challenge to provide adequate patient education about their disease, medication purpose, medication changes, reason for changes and side‐effects during short hospital stays. This suggests that more patient engagement is needed not only during hospitalization, but also in the discharge process and the period after hospitalization, especially for pharmaceutical care. This could be achieved by several methods, such as the use of lay language, asking patients what they want to know regarding their medicines, providing written information, repeating information or using the ‘teach‐back’ method, which is a strategy in which patients are asked to restate information that has been presented to them.^29^ As previous studies have shown that patients’ needs can increase after discharge, also a follow‐up phone call after discharge could be helpful to identify and to prevent medication‐related problems.^30^, ^31^, ^32^ Further research should find out how these interventions could help to reduce medication‐related readmissions.

The strength of this study is the description and comparison of the medication relatedness and potential preventability of readmissions according to perspectives of both patients and providers from several hospital departments. However, some limitations need to be discussed. This study is conducted in one hospital, which limits the generalizability. Another limitation is that patients were interviewed about the index admissions during readmission, which could lead to subjectivity and hindsight bias. However, in this way we could obtain information of the period after discharge of the index admission. Some patients could not be interviewed due to severe illness or unwillingness to participate. This could lead to selection bias as healthier or more satisfied patients were more often interviewed, which may have resulted in lower reporting of medication relatedness and preventability.

## 5 CONCLUSION

Patients and providers differ substantially on their perspectives regarding medication relatedness and potential preventability of hospital readmissions. According to patients, medication‐related readmissions occur more often and are more often potentially preventable compared with providers’ perspectives. Patients reported most often that actions on the hospital level were possible to potentially prevent the readmission. Further studies need to explore the reasons for the gap between patients’ and providers’ perspectives.

## CONFLICT OF INTEREST

None.

## Data Availability

Data are available on request from the authors.
