# Supplementary material for: Patients’ and providers’ perspectives on medication relatedness and potential preventability of hospital readmissions within 30 days of discharge
Source: Health Expect. 2019 Nov 16;23(1):212–9. doi: 10.1111/hex.12993 (PMC6978863; doi:10.1111/hex.12993)
Supplement: Supplementary file 1 [file HEX-23-212-s001.docx]

**Supplementary file - Interview**

Admission number:_______________________________________________________________________________

Date of interview:________________________________________________________________________________

Name of interviewer:_____________________________________________________________________________

**Questions for the patient:**

1. You were admitted to the hospital from______ to ______ Why were you admitted to the hospital? ________________________________________________________________________________________
2. What do you think that the reason is that you are readmitted?

________________________________________________________________________________________

Interviewer: I would like to discuss your experiences about your previous hospitalization from______ to ______

1. How much information did you receive during hospitalization on medicines you had to take at home?

a. No information

b. Some information, but not enough

c. As much information as I needed

d. Not applicable (I don’t take medication)

1. How much information did you receive during hospitalization on side effects of medicines you had to take at home?

a. No information

b. Some information, but not enough

c. As much information as I needed

d. Not applicable (I don’t take medication)

1. Did you receive written instructions upon discharge on medicines you had to take at home? If so, did anyone take the time to explain the written instructions?

a. I have not received written instructions

b. Yes, I have received written instructions but nobody has taken the time to explain them

c. Yes, I have received written instructions and yes, someone has taken the time to explain them

d. Not applicable (I don’t take medication)

Interviewer: I would like to discuss your experiences about the period between your previous and your current hospitalization

1. How many different medicines do you use at home? ________________________________________________________________________________________
2. Do you also use medicines that you buy without a prescription at a drugstore, pharmacy or health food store? (e.g. vitamins, anti-pain agents, soothing agents, herbs, homeopathic remedies, purchased online)

No Yes, namely _______________________________________________________________________

1. Are you receiving any help with your medication at home?

Yes, home care

Yes, family member or _____________ ______________________

Yes, week box / multi dose drug dispensing system

No

Not applicable (I don’t take medication)

1. Do you think your medication contributed to having to go to the hospital again? (e.g. side effect, too many medicines)

No Yes, namely _______________________________________________________________________

Not applicable (I don’t take medication)

1. If you look back at your last admission, is there something that your doctor, the hospital, your family or yourself could have done differently so that you would not have been admitted to the hospital again?

No Yes, namely

General practitioner _____________________________________________

Hospital _______________________________________________________

Family _________________________________________________________

Self ___________________________________________________________

Other _________________________________________________________

Interviewer: I would like to ask you some general questions.

1. What is your living situation at the moment?

I live alone I live together

1. In which country were you born?

The Netherlands Suriname NL Antilles Turkey Morocco Other, namely: ____________

1. In which country was your mother born?

The Netherlands Suriname NL Antilles Turkey Morocco Other, namely: ____________

1. In which country was your father born?

The Netherlands Suriname NL Antilles Turkey Morocco Other, namely: ____________

1. What is your highest completed education?

No training completed

Primary school

Secondary general education (such as MAVO, (M) ULO)

(Lower) vocational education (such as LTS, MBO, MTS, MEAO, MHNO, INAS)

Higher general secondary education (such as HAVO, VWO, HBS, MMS)

Higher professional education (such as HBO, HTS, HEAO, PABO)

University

Other, namely ……………………………………………

1. In general, would you say your health is:

a. Excellent

b. Very good

c. Good

d. Fair

e. Poor

**To be completed by the Interviewer:**

The questionnaire was completed with:

Patient himself Patient / caregiver Caregiver

Have you experienced a language barrier with the patient / caregiver:

No Yes, namely __________________________________________________________________

How long did the interview last in minutes: _________________________________________________

Remarks / details / notes

No Yes, namely ___________________________________________________________________
