# Supplementary material for: Patients’ and providers’ perspectives on medication relatedness and potential preventability of hospital readmissions within 30 days of discharge
Source: Health Expect. 2019 Nov 16;23(1):212–9. doi: 10.1111/hex.12993 (PMC6978863; doi:10.1111/hex.12993)
Supplement: Supplementary file 2 [file HEX-23-212-s002.docx]

|  | **Patient** | | | **Provider** | | | |
| --- | --- | --- | --- | --- | --- | --- | --- |
|  | **Patient's reason for readmission** | **Patient’s explanation that medication caused a potentially preventable readmission** | **Classification** | **Providers' reason for readmission** | **Providers' medication related** | **Providers' potentially preventable** | **Providers' explanation** |
| 1 | CVA | Dosage of blood pressure pill too high already mentioned at index admission (blood pressure was much too low) | DOSAGE | Recurrence of CVA | No | n.a. | Worsening of disease, despite treatment according to guideline |
| 2 | Stomach pain, shortness of breath | Too few pills to get INR at good value | DOSAGE | Shortness of breath | No | n.a. | Shortness of breath due to hyperventilation |
| 3 | Too few white blood cells | Prednisolone phased out too quickly | DOSAGE | Recurrence of Thrombotic thrombocytopenic purpura (TTP) | No | n.a. | Worsening of disease, despite treatment according to guideline |
| 4 | Clostridium infection | Vancomycin was stopped too early | DOSAGE | Aspiration pneumonia | No | n.a. | Worsening of disease, despite treatment according to guideline |
| 5 | Appendicitis surgery complication | Antibiotics should have been given some more days | DOSAGE | Stomach pain and nausea | No | n.a. | Calculated risk, treatment during index admission was in accordance with guidelines |
| 6 | Outages of the left brain | Dosage of dexamethason should have been higher | DOSAGE | Outages of the left brain | No | n.a. | Worsening of disease, despite treatment according to guideline |
| 7 | Shortness of breath | The anti-inflammatory medicines should have been given longer, then the cough/shortness of breath might not have come back | DOSAGE | STEMI | No | n.a. | Unlikely that STEMI is caused by medication |
| 8 | Shortness of breath | My inhalation medication should not have been stopped during index admission and prednisolone dosage was too low | MEDICATION CHANGE and DOSAGE | COPD exacerbation | No | n.a. | Worsening of disease, despite treatment according to guideline |
| 9 | CVA | Blood thinners were discontinued during index admission. | MEDICATION CHANGE | CVA | No | n.a. | Worsening of disease, despite treatment according to guideline |
| 10 | Shortness of breath | Confusion about diuretics, one was started and one was stopped | MEDICATION CHANGE | Heart Failure | Yes | No | Furosemide was stopped, this was a calculated risk. |
| 11 | Low blood pressure and pain in the calves | I have mixed up Oxynorm® and Oxycontin® | MEDICATION CHANGE | Worsening of infection and overall malaise | No | n.a. | Worsening of disease, despite treatment according to guideline |
| 12 | Lots of coughing and tired | Medication was out of stock in the pharmacy and the dosage was unclear. I needed more information and answers to my questions | MEDICATION CHANGE | Upper respiratory tract infection | No | n.a. | Worsening of disease, despite treatment according to guideline |
| 13 | Anxiety, could not stand up anymore | I could not pay my benzodiazepines at discharge and a lot of medication changes in my medication for depression could have caused my readmission. The hospital should not give me medication that I cannot pay. | MEDICATION CHANGE and COSTS | COPD exacerbation | No | n.a. | Worsening of disease, despite treatment according to guideline |
| 14 | High blood pressure | I discontinued indapamide, because of adverse drug reactions; I should have had more information about side effects. | ADHERENCE | hypertensive urgency | Yes | Yes | Indapamide, non-adherence |
| 15 | Urinary Infection | Interaction with medication for thyroid gland; discharge was too early. | DRUG-DRUG INTERACTION | Vomiting | No | n.a. | Worsening of disease, despite treatment according to guideline |
| 16 | Could not walk, due to Achilles tendon | I read in the package leaflet that ciprofloxacine could cause pain in the Achilles tendon | ADVERSE DRUG REACTION | Tendonitis by ciprofloxacin | Yes | No | Ciprofloxacin, adverse drug reaction |
| 17 | Vomiting | Medication that works on the stomach, my stomach had to be checked before discharge | NON-SPECIFIC | Stomach upset | No | n.a. | Complication of surgery |
| 18 | Shortness of breath | All the medication together, I got too much stress at home after discharge, and I had do to activities that I couldn't. | NON-SPECIFIC | COPD exacerbation | Yes | Yes | Seretide, non- adherence |
| 19 | Fallen at home | Side effects of metformin and paracetamol | NON-SPECIFIC | NSTEMI | Yes | No | Trastuzumab, adverse drug reaction |
| 20 | Unwell during a outpatient check | Due to side effects of oxydon or too much supplements: I should have stayed longer at index admission, to recuperate | NON-SPECIFIC | Overall malaise | No | n.a. | Worsening of disease, despite treatment according to guideline |
| 21 | Black toe | Medication could have caused my black toe: more diagnostics were needed. | NON-SPECIFIC | Worsening of diabetic foot | No | n.a. | Worsening of disease, despite treatment according to guideline |
